# Distribution of HCV genotypes and HCV RNA viral load in hepatitis infected patients of Kolar region, Karnataka, India

**DOI:** 10.6026/97320630018387

**Published:** 2022-04-30

**Authors:** Devinder Kaur, K Prabhakar, Subhashis Das

**Affiliations:** 1 Sri. Devaraj Urs Medical College, SDUMC, Kolar, Karnataka, India

**Keywords:** Hepatitis C virus, genotype, infection, viral load

## Abstract

Decisions and disease progression prediction, understanding the distribution of the hepatitis C virus (HCV) genotype and its association with viral load is significant for treatment. Therefore, it is of interest to document the distribution pattern of HCV genotypes and their association with viral load among HCV infected patients in Kolar, Karnataka. Seventy-four HCV-positive patients and not on antiviral therapy were enrolled from R.L. Jalappa hospital in Kolar, Karnataka. Blood samples were taken and demographics were recorded. HCV RNA was isolated after plasma was separated. qPCR was performed to measure the viral load, and RT-PCR was performed to determine the genotype. Genotype 3 was the prevalent (n=11, 40.7%) followed by genotype 4 (n=8, 29.6%), 2 (n=6, 22.2%), 1 (n= 13.7%), and mix (n=1, 13.7%). The median viral load of genotype 3 was a 2,87,835 IU/mL (IQR 10, 780-3, 71, 66) , genotype 2 was 81,030 IU/mL (IQR 66,495-95,565), genotype 4 was 43, 410 IU/mL (IQR 38, 355-48, 465) belongs to viral load less than 8,00,000 IU/mL. The median viral load genotype 3 was a 1, 05, 19, 500 IU/mL (IQR 49, 37, 250-2, 36, 71, 500), genotype 2 was 2,55,99,000 IU/mL (IQR 2,00,10,000-32,725,500), genotype 4 was 1,67,40,000 IU/mL (IQR 1,45,50,000-17,493,000) belonging to viral load more than 8,00,000 IU/mL category. A correlation between genotype and viral load was observed (p =1.5x10-12), of which genotype 3 showed a high viral load. Thus, HCV genotypes 1 2, 3, 4, and mixed genotype was observed in the patients studied. HCV genotype was associated with viral load in patient plasma. This data finds use in the treatment and prevention of hepatitis C in Kolar, Karnataka.

## Background:

The hepatitis C virus (HCV) is a major human pathogen of blood borne origin with approximately 130 million (3%) people infected worldwide and about 3-4 million new cases each year (WHO), and with the most affected regions being Central and East Asia and North Africa [1]. In India the estimated prevalence of HCV infection is about 1- 1.9% although across various geographical regions in India variations have been reported in the literature [2]. The disease manifests in the form of acute infection and if left untreated, progresses to chronic hepatitis and then to liver diseases like cirrhosis and hepatocellular carcinoma (HCC) [3]. Exposure to infected blood and blood products is the cause of HCV transmission and primarily the spread of the disease is through blood transfusion, surgery, sexual intercourse, injection drug use, and tattooing [4-5]. Both pretreatment serum HCV RNA levels (viral load) and viral genotype were determined to be the two strongest markers of treatment efficacy in recent clinical trials. As a result, after a patient has tested positive for HCV, it is now essential that viral load and genotype testing be performed to examine whether the patient is appropriate for treatment and the length of treatment [6]. Genotypes 1, 2, and 3 are the most common worldwide, while other genotypes are restricted to specific geographical areas. HCV genotyping reveals information regarding the viral genome's variability, illness progression, and therapeutic approaches [7].The duration and response to treatment are also impacted by the viral load of the patient [8]. The treatment of HCV starts with the screening and management of alcohol use to prevent the progression of the disease to cirrhosis [9-10]. Early treatment could prevent the disease progression and subsequent transmission [11-12]. Interferon-alpha (IFN-α) has been the first pan-genotypic option since the early 1990s, with sustained virologicresponse (SVR) rates of 8%-21% [13]. Therefore, it is of interest to determine the distribution pattern of HCV genotypes and their association with viral load in plasma samples of HCV infected patients.

## Material and Methods:

### Subjects:

The study was conducted among 74 patients who tested positive for HCV and negative for HIV and HBV. All patients were not taking any antiviral therapy at the time of the study. Patients were recruited from the R. L. Jalappa Hospital and Research Centre in the department Microbiology, attached to Sri DevarajUrs Medical College Tamaka, Kolar. A semi-structured questionnaire was used to collect demographic data (age and sex). The study was conducted from August 2019 to March 2020 using convenient time-frame sampling. Patients were informed about the study and given their informed written consent to participate. The study protocol was approved by the institutional ethical committee of Sri DevarajUrs Medical College, Kolar.

### Sample collection and Molecular analysis:

Blood collection:

A blood sample of 3 ml was collected by venipuncture and stored in the sterile tubes containing ethylene diaminetetraacetic acid (EDTA) and each subject was tested positive for anti-HCV antibodies by using CLIA immunoassays and negative for HIV and HBV. Separated plasma was stored in aliquots at -80°C for further analysis.

### RNA extraction:

Viral RNA was extracted using QIAamp Viral RNA Mini Kit according to the manufacturer's instructions (# catalog number 52904 QIAamp Viral RNA mini kit, Qiagen, USA).

### qPCR viral load detection:

qPCR was performed for the detection of HCV RNA using the HCV-K-004 kit (Co Sara Diagnostics. Pvt . Ltd). The kit uses quantitative qPCR with Magnetic Induction Cycler - quantitative polymerase chain reaction (Mic-qPCR, biomolecular systems). The HCV viral load kit included reagents and a readymade master mix with Internal Positive Control along with 5 standards. The negative control was included in parallel for each batch of analysis. The single-step reverse transcription real-time PCR transcribes and amplifies conserved sequence of RNA from the 5'untranslated region of HCV genome was detected by using Quasar 670 dye (Q670). A human RNase P gene marker was used as an internal positive control to check the reliability of each reaction, and the CAL Fluor Red 610 dye was being used to detect inadequate samples (CF610) The qPCR reaction was performed by using the total reaction volume of 10 µl and the HCV viral load thermal cycling conditions were as follows: activation at 42°C for 2 min hold,70°C for 1 min hold, 95°C for 20 sec and 45 cycles of 95°C 15 sec and 55°C for 60 sec. The PCR product was detected by using Quasar 670 dye (Q670).

### HCV genotyping:

HCV RNA positive samples that had viral load > 1000 international units per milliliter (IU/mL) were genotyped using Geno Sens. HCV-genotype-HCV Genotypes 1/2/3/4 PCR kit (Corbett Research, Australia). Briefly, total volume reaction 10µL (Reagent 1: HCV Genotyping Super mix 7.5µL and Reagent 2:Mg. Sol HCV Genotyping 2.5µL) and then add 15µL RNA for amplification. The PCR reactions were analyzed in a Qiagen Rotor Gene-Q Cycler 5-plex HRM System with software version 2.3.1. The thermal cycles included first hold 50°C for 15 minutes i.e. cDNA, Second hold denaturation 95°C for 15 seconds, Annealing steps 55°C for 20 Seconds Extension step 72°C for 15seconds and 45 cycles of 95°C for 20 minutes and 95°C for 10 seconds, 62°C for 30 seconds, and 56°C for 40 seconds. The analysis was completed within three hours by following the instructions.

### Statistical analysis:

The data was processed in an Excel program and analyzed using IBM SPSS Statistics 20.0. Across gender and age categories, the frequency of HCV genotypes was evaluated, and the median viral load with inter quartile range (IQR). A high viral load was defined as a baseline HCV RNA ≥ 800,000 IU/mL.

## Results:

A total number of 74 HCV infected patients were recruited from the Medicine department of the R. L. Jalappa Hospital and Research Centre. The patients included were 55 (74%) males and 19(24%) females, and the mean age was 49.02±16.92 years. 27(36.4%) out of 74 patients were detected with the viral load and genotyping. As shown in Figure 1(see PDF), genotype 3 was the prevalent (n=11, 40.7%) followed by genotype 4 (n=8, 29.6%), 2(n=6, 22.2%), 1(n= 13.7%), and mix (n=1, 13.7%). Distribution of different genotype with respect to gender and age is shown in Table 1(see PDF). The median viral load of genotype 3 was a 2,87,835 IU/mL (IQR 10,780-3,71,66), genotype 2 was 81,030 IU/mL (IQR 66, 495-95, 565), genotype 4 was 43,410 IU/mL(IQR 38,355-48,465) belongs to viral load less than 8,00,000 IU/mL .The median viral load genotype 3 was a 1,05,19,500 IU/mL (IQR 49,37,250-2,36,71,500), genotype 2 was 2,55,99,000 IU/mL (IQR 2,00,10,000-32,725,500), genotype 4 was 1,67,40,000 IU/mL (IQR 1,45,50,000-17, 493,000) belonging to viral load more than 8,00,000 IU/mL category. Majority of patients (7/27, 25.9%) was in age of 22-41 years (Table 1 - see PDF). 9 of 20 male patients (45%) were genotype 3, followed by 2 (5/20, 25%), 4 (4/20, 20%), 1(1/20, 5%) and mix 1 (1/20, 5%). No genotype 1 and mix type-infected female patients were identified (Table 1 - see PDF). Here, we defined RNA copies of < 800,000 IU/mL as low viral load, while ≥ 800,000 IU/mL was high viral load (Table 2, Figure 2 - see PDF). Overall, a correlation between HCV viral load and genotype were observed (p = 1.5x10-12). The genotype 3 had a greater number of patients with high viral load (n = 18).

## Discussion:

In India, HCV genotypes show varied distributions in different geographic regions. In north India, HCV genotypes 1, 2, and 3 have been found majorly with genotype 3 being the predominant one.

Data from south India showed a high occurrence of genotype 1 followed by 3 [15]. In the present study, the endeavor was to establish a correlation between HCV genotypes and viral loads in patients from Kolar, Karnataka, a state in southern India. We concluded that there exists a likely correlation between genotypes and viral load accentuating the idea that future studies and research to prevent and treat HCV must revolve around type 3 HCV, without ignoring the HCV 1 and 2. It was also established in another study that shorter therapy schedules for genotype 3 HCV infected patients with low baseline viral load could attain an SVR (sustained virological response) as compared to those with a high viral load [16].

Data shows that genotype 3 had a greater number of patients with a high viral load. A supporting study was conducted in India in which it was also found that HCV 3 was the most commonly found type in India with 63.38% prevalence [17]. Not only in India but in other neighboring Asian countries like Nepal and Pakistan, the HCV 3a is in higher percentages [18].In Iran and Bangladesh the predominantly found genotype was 3a and 3b in most of the patients establishing the fact that the trend of the predominant HCV 3 extends to a major part of the Indian sub-continent also [19]. Data shows that majority of the patients were in the age group of 22-41 which is consistent with another study that concluded that most of the HCV cases were found to be in the age range of 30-40 [20].Another important observation was found in a very significant study which showed over 70% percent of infections in the Sindh region of Pakistan were reported due to the HCV 3 genotype providing a strong base to the study that we have carried out [7]. It is known that HCV genotype 3 and 1 accounted for approximately 95 percent of the HCV infection in Delhi and surrounding areas. Also, two atypical subtypes like 3i and 3f were identified [17].

## Conclusions:

The continued monitoring of HCV genotypes is essential for the optimum management of chronically infected patients. We report the viral load and genotypes for the first time in the Kolar region, Karnataka, India. Genotype 3 was associated with high viral load.Knowledge on circulating genotypes have implications on the future vaccine formulations.
